# COVID-19 and (gender) inequality in income: the impact of discretionary policy measures in Austria

**DOI:** 10.1186/s41937-022-00084-6

**Published:** 2022-02-05

**Authors:** Michael Christl, Silvia De Poli, Dénes Kucsera, Hanno Lorenz

**Affiliations:** 1 Fiscal Policy Analysis (B2 Unit), European Commission (JRC), Seville, Spain; 2 Agenda Austria, Vienna, Austria

**Keywords:** COVID-19, EUROMOD, Micro-simulation, STW, Automatic stabilizers, D31, E24, H24

## Abstract

This paper analyzes the impact of the COVID-19 crisis on household income in Austria, using detailed administrative labor market data, in combination with micro-simulation techniques that enable specific labor market transitions to be modeled. We find that discretionary fiscal policy measures in Austria are key to counteracting the inequality- and poverty-enhancing effect of COVID-19. Additionally, we find that females tend to experience a greater loss in terms of market income. The Austrian tax–benefit system, however, reduces this gender differences. Disposable income has dropped by around 1% for both males and females. By comparison, males profit mainly from short-time work scheme, while females profit especially from other discretionary policy measures, such as the one-off payment for children.

## Introduction

The COVID-19 crisis has had a severe impact on economies all around the world, with a corresponding effect on incomes; certain economies have been affected more severely than others. The impact of the crisis not only depends on the regional development of the pandemic, but also on the country-specific exposure to certain sectors that are more likely to be influenced.

Countries more exposed to tourism and to global value chains, are more dependent upon international development, while more closed economies might be less affected by the crisis. The Austrian economy is both, strongly dependent on tourism and closely linked to global value chains. Thus, the economic downturn in 2020 was greater than in other central European countries, with a drop in real GDP of 6.6%.

The COVID-19 crisis has had a significant impact on the job market in 2020, thus affecting incomes on a similar scale. Various lockdowns led to severe restrictions within companies operating in different sectors. As a result, the increase in unemployment was considerably greater than trends observed in past decades and crises. In 2020, unemployment levels in Austria rose to their highest since 1946, the unemployment rate (as defined by national authorities) reached 12% during the COVID-19 crisis in 2020 compared to 7.4% in 2019. Additionally, at the peak of the crisis in April 2020, almost 30% of those in employment were transferred to short-time work (STW), meaning that their working hours were reduced substantially.

In addition to the generous STW scheme (with a net replacement rate of up to 90%) which has already proven its worth in stabilizing the effect on income during the financial crisis of 2008/2009, the Austrian government implemented several discretionary policy measures to cushion the significant loss of income among households. Two one-off payments for the unemployed, as well as a special payment for families (depending on the number of children) were introduced as an additional measure to protect households’ incomes and stabilize consumption.

All these developments have had a severe impact on household incomes. Given that standard survey data to analyze the impact on household income, which is a very significant socio-economic factor, are usually not available, a detailed simulation of the impact on a micro-level is highly important, not only from an academic point of view, but also from the perspective of policy-making. Our work contributes to the literature on several levels, which is very fast-growing in relation to the impact of COVID-19.

Firstly, we simulate wage compensation schemes on a micro-level, using a novel methodology of labor market transitions, based on micro-simulation techniques.^1^ This allows us to transit individuals to both unemployment and short-time working schemes in standard models.

Secondly, we add to the discussion regarding the cushioning effect of discretionary policy measures. Our paper investigates how well policy responses are able to cushion income losses in Austria, and how inequality and poverty have been affected by the COVID-19 crisis. Our focus lies on household disposable income. We will mainly focus on direct labor market interventions, as income generated from employment is the most important element.

Hence, thirdly, our detailed administrative data, relating to the number of people in short-time work schemes and the scale of these schemes, allow us to model the duration and the reduction of working hours, to facilitate a more precise assessment of the impact of the COVID-19 crisis on the Austrian labor market. With an additional distinction between male and female employees in STW and unemployment, we assess whether the pandemic has resulted in additional gender differences.

We find that discretionary policy measures in Austria are key to mitigating the loss in income resulting from the COVID-19 crisis, especially with regard to poorer households. As a result, these measures are also crucial in fighting the inequality-enhancing effect of the COVID-19 crisis and in substantially offsetting the increase in poverty caused by the crisis. We also show that these discretionary policy measures help both males and females. However, males seem to profit more significantly from STW schemes, while females from other discretionary policy measures.

Our paper contributes to the literature in two ways. First, contrary to Almeida et al. (2021b) or Christl et al. (2021a) we are able to distinguish between different policy instruments. We differentiate between automatic stabilizers and discretionary policy measures. Within the discretionary policy measures, we focus on the impact of each single component: the STW schemes, the one-off payments for unemployed, as well as the one-off payment for families. Second, we introduce a counterfactual scenario that simulates a hypothetical scenario where STW schemes and other discretionary policy measures do not exist. We estimates the loss in working hours in the Austrian economy due to COVID-19 and move the corresponding amount of people to unemployment or social assistance. This allows us to get an intuition of the impact of the COVID-19 pandemic on household income in the absence of discretionary policy measures. Additionally, we are also able to analyze the effectiveness of those policy measures in stabilizing household income.

This paper is structured as follows: Sect. 2 briefly introduces the related literature, Sect. 3 describes the discretionary policy measures introduced, by the Austrian government to mitigate the income loss of households during the COVID-19 crisis. In Sect. 4, both the methodology and the underlying administrative data used are explained in detail. Section 5 presents the results, while Sect. 6 concludes.

## Literature review

The economic literature on the impact of COVID-19 is growing rapidly: many articles investigate the consequences of the virus on inequality in different countries. Clark et al. (2020) studied how inequality was affected during the COVID-19 crisis. Using survey panel data, they investigated income inequality in France, Germany, Italy, Spain and Sweden. They found that the pandemic effect in 2020 could be divided into two periods. With the exception of Germany, relative income inequality increased in the countries investigated during the first period (January to May); however, during the second period (by September), as a result of the effect of various policy interventions, the initial increase in income inequality had been reversed.

Almeida et al. (2021a) estimated the impact of the COVID-19 pandemic on household income within all EU Member States and the EU. They found that in 2020, disposable household income in the EU fell by around 9.3%. However, discretionary fiscal policy measures played a significant cushioning role, reducing the extent of income loss. They found that the average equivalent disposable income dropped by just 4.3%. They also estimated the significant effect of the tax–benefit systems in mitigating the impact of the pandemic on poverty and inequality; however, they also identified substantial differences across countries.

Similarly, Cantó et al. (2021) evaluate government policy responses in April 2020 in Belgium, Italy, Spain and the UK. Additionally, Christl et al. (2021a) estimate the impact of COVID-19 related policy measures in a cross-country setup for all EU Member States, showing that policy measures were cushioning substantially the income loss and the inequality increasing feature of the COVID-19 pandemic. Besides the differences in terms of automatic stabilization of the tax–benefit system to mitigate the effect on household income, caused by the COVID-19 pandemic, it was noted that COVID-19-related policy responses differ substantially across countries, leading to rather different impacts on the income protection mechanism.^2^

Several studies have analyzed the effect of STW during the crisis. STW prevents many short- and long-term effects on the labor market and consequently, on household or individual income. Stevens (1997) and Davis and von Wachter (2011) argue that loss of earnings resulting from job losses are considerably more persistent and severe, when these occur during a recession. Additionally, workers forgo returns to experience, which in turn, affects their employment prospects in the future [see, e.g., Jarosch (2015)].

Specifically in relation to the COVID-19 crisis, Christl et al. (2021b) investigated the impact of STW schemes on German household income, using a micro-level approach, combined with labor market transition techniques to simulate the effect of COVID-19 on the German labor market. The impact of the pandemic was found to be significantly regressive, with a detrimental impact on the poorest households, which was almost entirely offset by automatic stabilizers and discretionary policy measures. The STW schemes and especially the one-off payment for children were found to be an effective policy in terms of mitigating any income loss, particularly among the poorest families in Germany.

The effects of the COVID-19 crisis on men and women were found to be different from other economic downturns, as “standard” recessions mainly affected the economic sectors where men primarily work, whereas women work tend to work in non-cyclical sectors, such as health care or education. In the recent financial crisis in 2008, the job losses sustained by men were much higher than was the case for women. Since the current crisis has not affected not only industry, but also service occupations (accommodation and food service activities), the effect on job losses between genders has been more balanced. Nevertheless, school and daycare closures increased the need for childcare, which in turn, had a greater effect on mothers, rather than fathers.

Adams-Prassl et al. (2020) show that the first few months of the pandemic had a negative impact on labor-force participation and hours worked. These effects were found to be higher for less-educated workers and women, which exacerbated pre-existing inequalities. Using a simulation model, Alon et al. (2020) show that the impact of COVID-19 is likely to further increase gender inequality, by placing a disproportionate burden on women with additional childcare duties. Since the gender distribution of the labor force is different among economic sectors, additional gender differences arise due to the feasibility of working from home. Although widely studied, there is no clear evidence of the effect of working from home. While Bloom et al. (2015) and Arntz et al. (2019) find that working from home is likely to reduce (or at least not increase) wage differences between male and female workers, Goldin (2014) and Bertrand (2018) highlight results which prove the converse. In the current COVID-19 crisis, Bonacini et al. (2020) find that working from home is easier for older, better-educated and higher-paid male workers, thereby increasing labor-income inequality.

Palomino and Sebastian (2020) also estimate irregular wage losses and increasing wage inequality between males and females in four hypothetical scenarios regarding stringent policy responses (two months of lockdown, two months of lockdown plus six months of partial functioning of closed activities at 80%, 70% and 60%) across 29 European countries. However, they indicate that STW schemes are likely to compensate for the negative effect of the pandemic on increasing gender differences.

## COVID-19-related policy measures in Austria

In this section the main discretionary policies, introduced to fight the negative impact of the COVID-19 crisis on household income, are briefly described. All policies have been modeled in detail in the microsimulation model, EUROMOD, in order to estimate the impact of those policies on an individual and a household level.

### COVID-19 STW

In order to counteract the negative economic impact of COVID-19, the Austrian government introduced a new STW scheme in March 2020, the ‘COVID-19-Kurzarbeit’ [see, e.g., RIS (2020b)]. The program allows for a temporary reduction of normal working hours and remuneration. During the STW phase, employees enjoy job security and the employed person cannot be dismissed for an additional month after the period of short-time work comes to an end. In the case of terminations for personal reasons, the employer is obligated to employ a new employee.

Three phases of the program were introduced in 2020. During the first phase, COVID-19 STW was introduced retroactively for three months, starting on March 1, 2020. During this phase it was permitted to reduce normal weekly working hours by at least 10% (up to a maximum of 90%) averaged across the STW period. Within the scheme, flexible working time reduction also allowed for a temporary reduction of up to 100% during certain weeks, provided that the average working time over the whole period of STW was at least 10%. Employees were guaranteed up to 90% (up to 1700 EUR gross monthly salary), 85% (between 1700 EUR and 2685 EUR gross monthly salary) or 80% (above 2685 EUR gross monthly salary) of their former net income, regardless of the extent of work reduction. No STW compensation was paid for any portion of salary with a gross monthly income in excess of 5370 EUR.

The policy was extended for a further three-month period under the same conditions in the second phase. Should STW be requested for a further three months, employees would have to use three weeks of their vacation from the current vacation year (if they had accrued the sufficient number of days).

In the third phase of COVID-19 STW, new admissible minimum and maximum working hours were introduced. The previous minimum threshold was increased from 10% to 30%. In special cases, minimum working hours below this limit could be agreed (especially during the second lockdown period which began in November). The maximum working hours were adjusted to no more than 80%. Under new rules, it was mandatory for employees to attend additional training events during quiet periods, resulting from STW. The additional training had to be agreed with the Public Employment Service Austria and could commence at any time; it could be interrupted in accordance with the employer’s labor requirements and taken up again within a period of 18 months.

### One-off payment for the unemployed

In addition to short-time work, another important discretionary policy measure was introduced, to mitigate the consequences of the pandemic: a one-off payment for the unemployed. Two separate one-time payments were introduced for eligible unemployed persons in 2020 [see, e.g., RIS (2020a)]. The first one-off payment was introduced during the period between May and August. Any person, who was registered as unemployed for at least 60 days during this period, received a one-off payment of 450 EUR. Similarly, the second one-off payment provided 450 EUR in addition to the unemployment benefit, if the period of unemployment lasted for at least 45 days between September and November. This allowance was gradually reduced according to the duration of unemployment: 300 EUR for a period of unemployment lasting between 30 and 45 days and 150 EUR for a duration of unemployment of between 15 and 30 days. Both payments were also introduced for recipients of unemployment assistance.

### Special payment for families

Families with children received a special COVID-19-related one-off payment in September 2020. Every household with children received an additional payment of 360 EUR with their family allowance for every child living in the household [see, e.g., RIS (2020c)].

## Methodology and data

### Methodology

To assess the impact of a severe crisis, such as the COVID-19 crisis, detailed information on household income is required. Due to the lack of up-to-date survey data, several different methods are used to forecast the impact of profound effects on the labor market at a micro-level. In the literature, two approaches are typically discussed [see, e.g., Gasior and Rastrigina (2017)]: re-weighting and modeling labor market transitions.

Re-weighting of the underlying micro-data can be used to adjust the micro-data to up-to-date macro-data. This approach has the advantage of accounting not only for changes in the labor market, but also for changes in the labor market structure. So far, several papers, such as Almeida et al. (2021a) and Li et al. (2020), have taken advantage of this modeling approach to estimate the impact of the COVID-19 crisis on household income, as well as its related indicators, such as the Gini index (income inequality) and poverty.

However, as argued by, e.g., Gasior and Rastrigina (2017) or Cantó et al. (2021), this approach has certain shortcomings. Firstly, the new pool of unemployed is assumed to have similar characteristics to that observed in the data, an assumption that can be disproved during the COVID-19 crisis, since its effects was driven by several lockdown measures and certain sectors were more severely impacted than others. Secondly, as regards the re-weighting approach, a detailed simulation of compensation schemes (such as STW schemes) cannot be directly taken into account. Thus, the potential heterogeneity across the income distribution of such schemes also cannot be accounted for.

Therefore, other papers, such as Christl et al. (2021a), Christl et al. (2021b), Cantó et al. (2021), Brewer and Tasseva (2020) and Figari and Fiorio (2020) have simulated adjustments to the underlying micro-data, using microsimulation techniques to model labor market transitions. The basic idea is to model transitions from employment to both unemployment and other compensation schemes (such as STW schemes). Given specific individual information, both the hypothetical unemployment benefit and wage compensation can be simulated and individual benefits can be estimated. This approach enables all micro-data to be updated, using all available information.

In this paper, we follow exactly this approach. We use detailed data of the EU Statistics on Income and Living Conditions (EU-SILC) in combination with EUROMOD^3^ to simulate the whole tax–benefit system of Austria. The version used is based on the policy year of 2020, combined with input data from EU-SILC 2018. Market income variables and non-simulated benefits are uprated to 2020, using specific uprating factors.^4^ Labor market changes related to COVID-19 are simulated, using up to date detailed administrative data on the number of persons becoming unemployed and moving to STW schemes. This information allows us to replicate labor market changes by moving individuals from one state to another.

We then adjust the labor market characteristics and income of each individual, which changes the latter’s labor market status on micro-level. Additionally, we simulate the variables needed for the simulation of unemployment benefits (such as previous work history, previous wages, duration) and STW schemes (such as hour reduction, previous wages, duration). These adjustments are performed using the Labor Market Adjustment (LMA) add-on, which is a EUROMOD tool that can be used to simulate labor market transitions to employment, unemployment and monetary compensation schemes. The detailed description of the add-on can be found in the technical annex of Christl et al. (2021a).^5^ Using EUROMOD, we can then recalculate the whole tax–benefit system, taking into account the new labor market status of individuals that have been observed as a result of the impact of COVID-19.

To identify those that transit to wage compensation schemes and unemployment, we use detailed information from the Public Employment Service Austria (AMS). These administrative data not only facilitate a detailed view of specific sectors, often argued to be a main driver of the unequal impact of the COVID-19 pandemic, but also an analysis of gender. Detailed information on the data will be discussed in Sect. 4.2.1.

#### Definition of simulation scenarios

Following Christl et al. (2021b), we base our analysis on the comparison of three different scenarios: firstly, a baseline scenario that is the 2020 policy scenario, not including the effect of COVID-19 and not including the impact of the pandemic on the labor market. Secondly, we consider a COVID-19 scenario that not only includes the simulation of related discretionary policy measures, but also the COVID-19-related adjustment of the labor market (transitions to both unemployment and STW schemes according to external, administrative information). Thirdly, we create a counterfactual scenario, in which we assume the COVID-19-related labor market shock; however, we assume the absence of the COVID-19-related discretionary policy measures. This allows us to estimate the impact of discretionary policy measures in mitigating the effect on household income during the COVID-19 crisis.

Let *f* be the tax–benefit function that depends on the tax–benefit structure (the specific policy rules in place), *P* as well as on the status of the labor market *LM*. We assume that the policy rules *P* can either constitute the standard rules that were in place before COVID-19, the so-called automatic stabilizers, $P^{\mathrm{AS}}$, or can include the discretionary policy measures $P^{\mathrm{COVID}}$. The labor market condition, LM, can either be a scenario without COVID-19-related changes affecting the labor market (${\mathrm{LM}}^{{\mathrm{NoTrans}}}$) or with COVID-19-related labor market transitions (${\mathrm{LM}}^{{\mathrm{Trans}}}$).

Therefore, we can define our three scenarios as follows:

- *Baseline scenario:* $$f(P^{{\mathrm{AS}}}_{2020}, {\mathrm{LM}}^{{\mathrm{NoTrans}}}_{2020})$$.
- *COVID-19 scenario:* $$f(P^{{\mathrm{Covid}}}_{2020}, {\mathrm{LM}}^{{\mathrm{Trans}}}_{2020})$$
- *Counterfactual scenario:* $$f(P^{{\mathrm{AS}}}_{2020}, {\mathrm{LM}}^{{\mathrm{Trans}}}_{2020})$$

Please note that in the counterfactual scenario, we assume that instead of entering into compensation schemes, people would only have access to the traditional automatic stabilization mechanisms, such as unemployment benefits. In this scenario, we assume the same loss in terms of hours worked, as in the COVID-19 scenario. However, the impact affects less people, since individuals that become unemployed reduce their working hours to zero, while under STW schemes, individuals can reduce their working hours to a certain level (retrieved from external data).

To estimate the direct COVID-19 effects ${\mathrm{PE}}^{{\mathrm{Covid}}}$ in 2020, we consider the changes between the first two scenarios, focusing on both changes in the labor market and policy changes (responses):

1 $$\begin{aligned} {\mathrm{PE}}_X^{{\mathrm{Covid}}}=X\Bigl (f\bigl (P^{{\mathrm{AS}}}_{2020},{\mathrm{LM}}^{{\mathrm{NoTrans}}}_{2020}\bigr )\Bigr )-X\Bigl (f\bigl (P^{{\mathrm{Covid}}}_{2020},{\mathrm{LM}}^{{\mathrm{Trans}}}_{2020}\bigr )\Bigr ) \end{aligned}$$

The function *X* can either constitute a certain income concept (disposable income or market income), but also indicators such as the AROP or the Gini coefficient.

We then define the policy effects of the traditional automatic stabilizers (in the absence of discretionary policy measures) related to a function, *X*, as the difference between the first and the third scenario.

2 $$\begin{aligned} {\mathrm{PE}}_X^{{\mathrm{AS}}}=X\Bigl (f\bigl (P^{{\mathrm{AS}}}_{2020},{\mathrm{LM}}^{{\mathrm{NoTrans}}}_{2020}\bigr )\Bigr )-X\Bigl (f\bigl ((P^{{\mathrm{AS}}}_{2020}, {\mathrm{LM}}^{{\mathrm{Trans}}}_{2020}\bigr )\Bigr ) \end{aligned}$$

Comparing the two policy effects (${\mathrm{PE}}_X^{{\mathrm{AS}}}$ and ${\mathrm{PE}}_X^{{\mathrm{Covid}}}$) allows us to gain an insight into the impact of STW and other discretionary policy measures.

#### Automatic stabilization coefficient

In crisis times, automatic stabilizers as well as discretionary policy measures play a central role in cushioning household income. To assess the income stabilizing effect of the Austrian tax–benefit system, as well as any of its individual components, we follow the approach of Dolls et al. (2012) that was also employed by Christl et al. (2021a) in a cross-country set up and by Christl et al. (2021b) for Germany and defines the Income Stabilizing Coefficient (ISC) as:

3 $$\begin{aligned} {\mathrm{ISC}} = 1 - \frac{\sum _i \Delta Y^{D}_{i}}{\sum _i \Delta Y^{M}_{i}} = \frac{\sum _i \Delta Y^{M}_{i} - \sum _i \Delta Y^{D}_{i}}{\sum _i \Delta Y^{M}_{i}} \end{aligned}$$

where $\Delta Y^{D}_{i}$ is the disposable income change of an individual *i* and $\Delta Y^{M}_{i}$ is the change in the market income of the individual *i*. An ${\mathrm{ISC}}=0.8$ would imply that 80% of the effect on market income is absorbed by the tax–benefit system.

Following this approach, we can further decompose the effect of several tax–benefit instruments, such as taxes, social security contributions and benefits, which are typically called automatic stabilizers. Additionally, and of special interest, is an analysis of the impact of discretionary policy measures (such as short-time work and other measures, e.g., the aforementioned one-off payments for the unemployed) on the automatic stabilization mechanism of the tax–benefit system.

We, therefore, define discretionary policy measures ${\mathrm{DPM}}_i$ as the sum of the benefit of STW ${\mathrm{STW}}_i$, the two one-off payments for the unemployed ${\mathrm{BUN}}_i^{{\mathrm{OOP}}}$, as well as the one-off payment for children ${\mathrm{BCH}}_i^{{\mathrm{OOP}}}$:

4 $$\begin{aligned} {\mathrm{DPM}}_i={\mathrm{STW}}_i+{\mathrm{BUN}}_i^{{\mathrm{OOP}}}+{\mathrm{BCH}}_i^{{\mathrm{OOP}}} \end{aligned}$$

We then further decompose the ISC:

5 $$\begin{aligned} {\mathrm{ISC}} = \frac{\sum _i \Delta Y^{M}_{i} - \sum _i \Delta Y^{D}_{i}}{\sum _i \Delta Y^{M}_{i}} = \frac{\sum _i \Delta T_{i} + \Delta {\mathrm{SIC}}_{i} - \Delta {\mathrm{BEN}}_{i} - \Delta {\mathrm{DPM}}_{i}}{\sum _i \Delta Y^{M}_{i}} \end{aligned}$$

where, $T_i$ are taxes, ${\mathrm{SIC}}_i$ social insurance contributions, ${\mathrm{BEN}}_i$ benefits, ${\mathrm{DPM}}_i$ are all the discretionary policy measures paid or received by an individual *i*. Following this notation, we are able to decompose the income stabilization to the specific tax–benefit instruments.

### Data

#### Administrative data for unemployment and STW

**Fig. 1:**
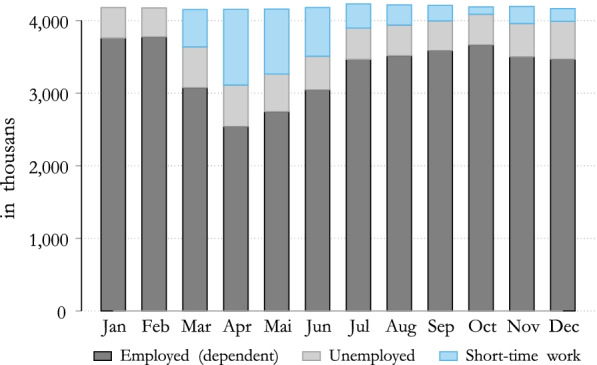
Labor force in the first year of the COVID-19 crisis. Note: Data available on 2/2021. Source: Own calculation, Public Employment Service Austria (AMS).

In order to evaluate the effect of a transition to STW or to unemployment, the labor market status of all individuals are adjusted, using monthly data from the Public Employment Service Austria (AMS). The simulation of the STW is based on the data available from March until December 2020, and takes into account information relating to the number of people in STW, the normal working hours and the reduction of working hours as a result of STW across sectors and gender. As an immediate impact of the COVID-19 crisis, unemployment increased. Figure 1 highlights that the first few months of the crisis, in particular, were critical: in April almost 600,000 people were registered as unemployed and almost twice as many were on STW. Therefore, more than 1.5 million people or almost 40 percent of the labor force, were either unemployed or in STW. Hence, STW has succeeded in limiting the impact of the COVID-19 crisis on the labor market and on unemployment.

Due to the lockdown and official closures, as well as the unequally distributed home-office possibilities, certain sectors have been more significantly affected that others. To capture this effect in the simulation of labor market transitions, we include detailed information in our estimates relating to the use of STW by sector.

**Fig. 2:**
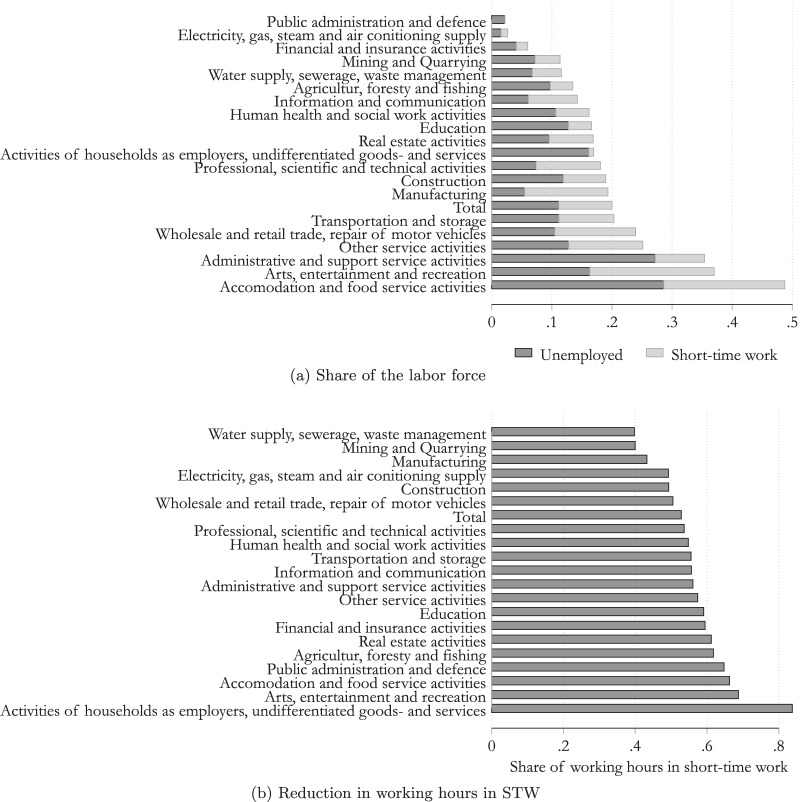
The impact of the COVID-19 crisis on the labor market by sector in 2020. Note: Data available on 2/2021. Average for the period March to December 2020. Source: Own calculation, Public Employment Service Austria (AMS).

Figure 2a highlights that from March until the end of the year 2020, more than half of the labor force within the sector, “accommodation and food service activities”, were either unemployed or in STW schemes. Focusing on the date, the effect of the first lockdown was greatest in this sector. At the end of April, more than 90% of people in this sector were either unemployed or in STW.

The utilization of short-time work differed substantially across sectors. More than every fourth employee worked to a limited extent in the areas of “accommodation and food service activities” and “arts, entertainment and recreation”, but STW was also used to a great extent in the sectors with the highest numbers of employees, namely the “wholesale and retail trade”, “repair of motor vehicles” and “manufacturing”. Our model will take these detailed sectorial differences into account.

Detailed administrative data allow us to calculate the share of the reduction in working hours, as highlighted in Fig. 2b and in Table 3 in the Appendix. This shows that the reduction in working hours peaked during the months of lockdown. During the period from March to December, the average reduction in working hours was equal to 53%. Nevertheless, the pattern in the reduction of working hours indicates huge differences across sectors. The reduction in working hours was highest in the sectors “arts, entertainment and recreation” and “accommodation and food service activities”, recorded in excess of 66%, while the sectors “mining and quarrying” and “water supply, sewerage, waste management” reported the lowest figures at less than 40%.

**Fig. 3:**
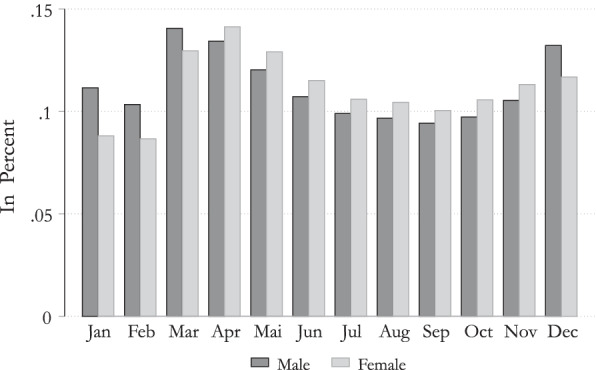
Unemployment rate by gender in 2020 Source: Own calculation, Public Employment Service Austria (AMS).

Due to general gender differences in employment by sector and the fact that certain sectors have been more significantly affected than others, our paper will also shed light on the gender differences in unemployment and STW, and the consequences on income. The gender difference in unemployment rate is highlighted in Fig. 3. We can see that before the COVID-19 crisis hit the Austrian labor market, the unemployment rate was slightly higher for males than for females. This, however, changed when first lockdown measures were introduced and general unemployment increased. While in February the unemployment rate of females was about 8% and the one for males about 10%, in April the rate increased to about 13% for males and 14% for females.

**Fig. 4:**
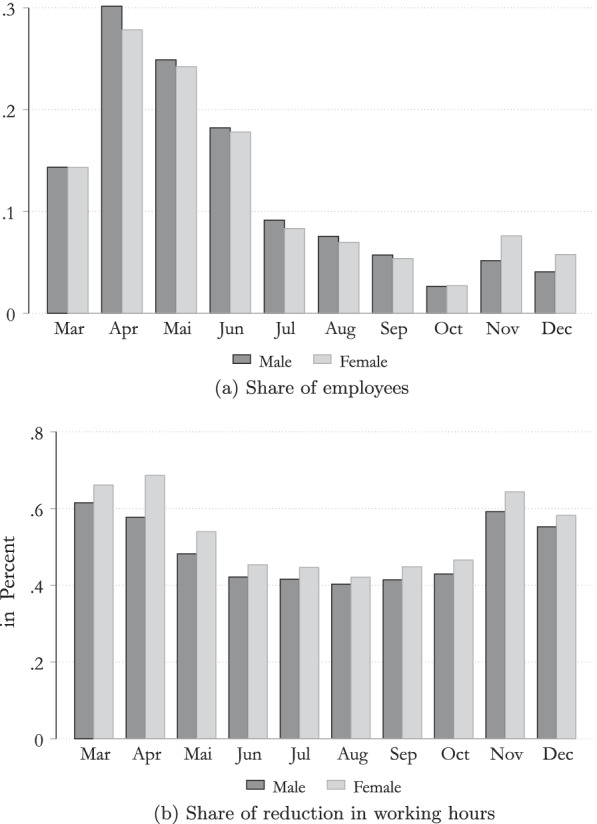
Differences in STW schemes by gender in 2020. Note: Data available in 2/2021. STW available in the year 2020 only, from March till December. Source: Own calculation, Public Employment Service Austria (AMS).

On the other hand, when looking at STW, administrative data reveal that the relative share of employees in STW (as a fraction of the number of employees) was higher for in the case of male employees at the beginning of the COVID-19 crisis. Nevertheless, since October female employees are on average slightly more likely to be in STW, compared to their male colleagues, as highlighted in Fig. 4a. During the last year, these two effects therefore, almost cancel one another out. On the other hand, Fig. 4b shows that there are gender differences in relation to the average reduction in working hours in STW. The reduction was greater in the case of female employees each month since the COVID-19 STW was introduced.

#### Estimating the duration of STW

There is no information in the administrative data for how long individuals stayed in STW schemes. We therefore set up a model based on survival probabilities to obtain estimates for the duration of STW scheme. As shown in Fig. 4a, we have detailed information on the number of individuals in STW in each month. This information is available by gender and each sector of activity. The STW started in March, and we assume that the total number of people entering in STW over the year is reached in the month with the highest share of people (April 2020). During the following months, we assume that some people managed to go back to work and no new persons entered into the scheme. Due to the second wave of COVID-19, there is a slight increase in the number of persons in STW in November and December. We assume that people entering in the schemes in November and December are employees who were already in STW in previous months.

To estimate the duration in STW, we sort the months in a descending order, based on the number of persons in STW in each month. This allow us to estimate the probability to go back to work in each month. Using these probabilities, we estimate the share of people staying from 1 up to 10 months, by sector of activity and gender.^6^

**Fig. 5:**
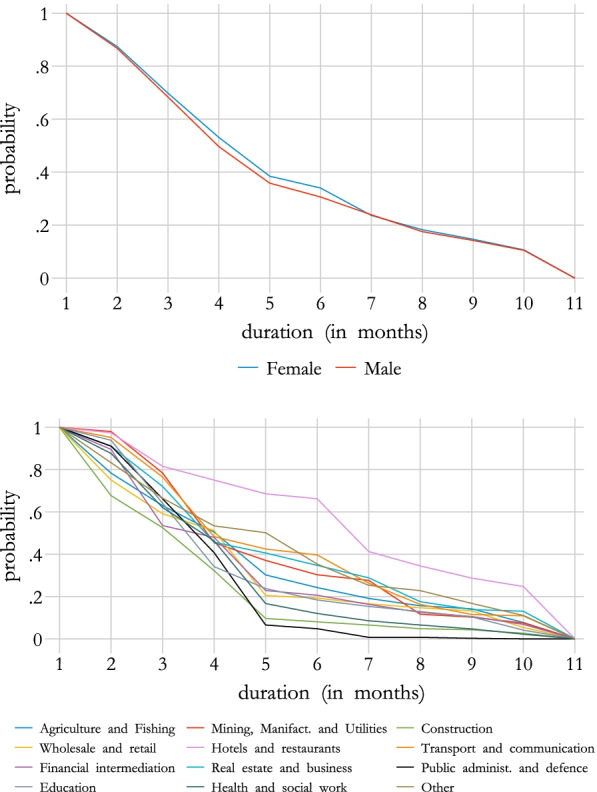
Estimation of duration in STW, by gender and sector Source: Own calculation, Public Employment Service Austria (AMS).

Figure 5 shows the duration in STW of people that moved to short-time work by gender and sector. We see that related to the duration, there are no big differences across gender. However, we find substantial differences across sectors, highlighting the importance of taking sector specific information into account. While the duration for people working in public administration was on average very short (more than 90% of workers stayed 5 months or less in STW), workers in the hotels and restaurants were on average very long in STW (almost 25% of them stayed the full 10 months of 2020 in STW schemes).

## Results

In this section, we will first discuss the impact of both the changes in the labor market (a substantial loss in hours worked in the economy) and the policy responses to the COVID-19 crisis, according to which people were able to move to STW and were eligible for additional discretionary policy measures that were implemented to mitigate income loss due to labor market changes.

In the second section, we will consider the impact on standard inequality and poverty measures that will allow us to evaluate the impact on standard indicators, often used to analyze the socio-economic impact of crises and policy instruments. Finally, in the third section, we will take a closer look at gender differences with regard to the direct impact of the COVID-19 crisis, as well as specific policy responses.

### The COVID-19 impact and the mitigating effect of discretionary policy measures

Firstly, we will focus on the drop in income across the income distribution. We distinguish between the impact on original (market) income, as well as disposable income. We define the difference as both the policy effect and the mitigating effect of the tax–benefit system set against the loss of household income.

**Fig. 6:**
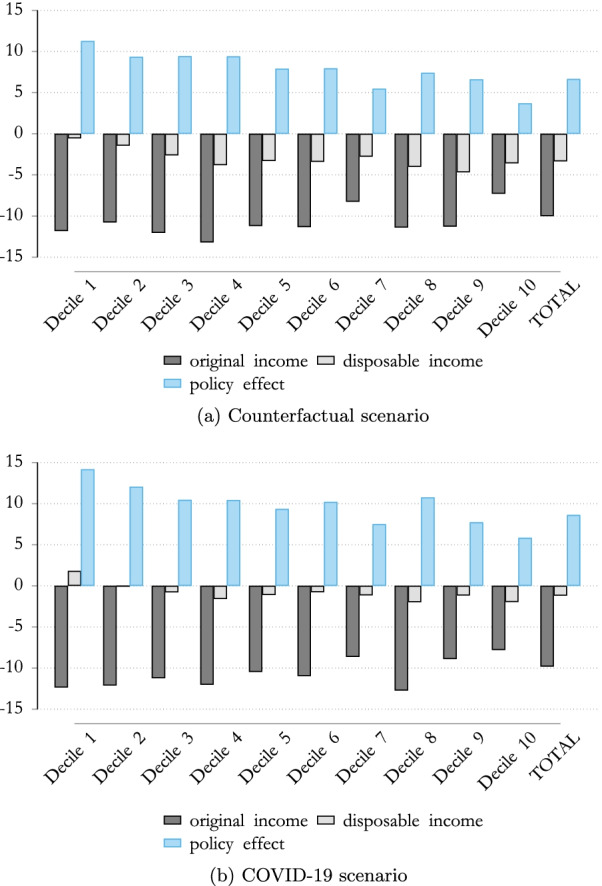
The impact of COVID-19 on household income Note: Percentage change in equivalized original and disposable income compared to the baseline scenario by income deciles. Income deciles are based on the baseline scenario distribution of equivalized disposable income. Source: Own calculations, based on EUROMOD I3.0+

Figure 6 highlights these concepts with regard to the Austrian population, in both the counterfactual scenario and in relation to the COVID scenario. Focusing on the impact of the COVID-19 crisis on original income, we can see that in the absence of discretionary policy measures (counterfactual scenario), original incomes dropped substantially, especially in the case of lower incomes, where the drop in original income was around 10% to 13%. However, the standard automatic stabilizers were able to offset this negative income effect, at least in relation to those at the lower end of the income distribution, where this drop in disposable income was mitigated almost completely. Figure 6a shows that without discretionary policy intervention, the tax–benefit system substantially absorbed the COVID-19 shock and even demonstrated a slightly progressive impact, with employees on lower incomes profiting more in relative terms, than those on higher incomes.

Taking into account discretionary policy measures, discussed in detail in Sect. 3, the mitigating effect on the loss of household income, as a result of the tax–benefit system, changed substantially. The impact on original income would, on average, be the same (around − 10%) in both scenarios; however, this differed slightly across the income distribution (due to the fact that the reduction in total hours worked can be shared among workers, and therefore, firms can adjust labor demand more flexibly), as highlighted in Fig. 6b. We can see, however, that the drop in disposable income is substantially lower when taking into account the discretionary policy measures. Additionally, we observed that discretionary policy measures, such as the STW schemes and the one-off payments, more than offset the income loss in the case of low income earners, leading to an increase in disposable income in the first and second decile.

The mitigating effect on household income (policy effect) of the tax–benefit system that also includes discretionary policy measures is substantially higher than in the counterfactual scenario and slightly more progressive.

**Fig. 7:**
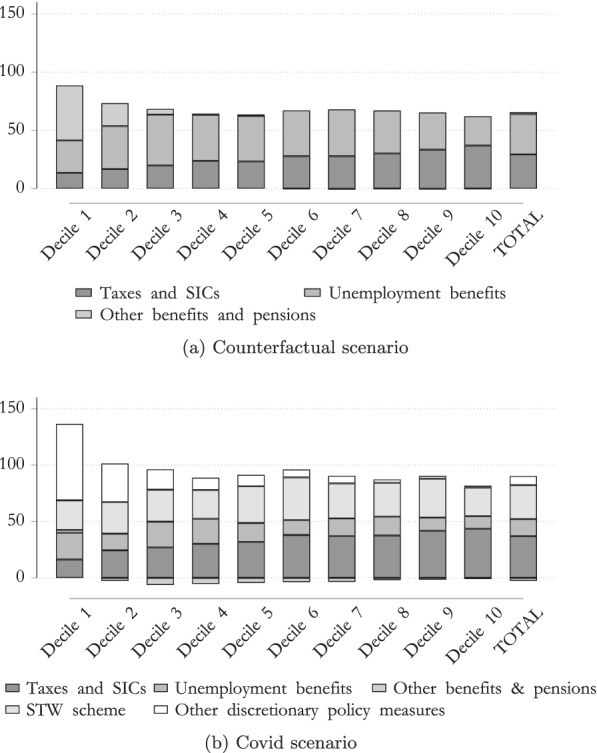
ISC in Austria.Note: Percentage change in equivalized original and disposable income compared to the baseline scenario by income deciles. Income deciles are based on the baseline scenario distribution of equivalized disposable income. Source: Own calculations, based on EUROMOD I3.0+.

To see the impact of different policy instruments, we will take a closer look at the ISC of the tax–benefit system and its decomposition. Figure 7 highlights the differences of the ISC by deciles and by tax–benefit component. It has been noted that the discretionary policy measures (that are included in the COVID scenario) lead to a substantial increase in the automatic stabilization mechanism of the tax–benefit system. In other words, these policy measures are crucial in terms of absorbing the income shock in the Austrian tax–benefit system, especially at the lower levels of the income distribution.

While the automatic stabilizers of the Austrian tax–benefit systems absorbed around 88% of the lowest income decile and around 65% of the highest income decile, discretionary policy measures also helped mitigate the loss of income in the lower income distribution, as highlighted in Fig. 7a. It is especially interesting that in the lowest two deciles the one-off payments for the unemployed, as well as for children (white bar) were crucial policy measures in mitigating income loss, while STW schemes played only a minor role. The effect of STW schemes, however, was particularly important in mitigating income losses in relation to the middle income distribution, in which the decrease in original (market) income was almost entirely offset by the tax–benefit system with its discretionary policy measures.

### The COVID-19 impact on inequality and poverty

Having considered the mitigating effect on the loss of income across the income distribution, we now focus on several concepts related to inequality and poverty. Again, we concentrate on the impact of COVID-19 on inequality in the absence of discretionary policy measures (counterfactual scenario) and vice versa (COVID-19 scenario).

As highlighted in Table 1, in both the counterfactual scenario (CF) and the COVID-19 scenario (COVID), inequality in terms of original (market) income, as measured by the Gini index increased, highlighting that COVID-19 had an inequality-enhancing character, when focusing on original income, irrespective of the scenario used. The Gini index increased by 3.7pp in the CF scenario and by 2.3pp in the COVID scenario.

**Table 1:** The impact of COVID-19 on inequality (Gini). Source: Own calculations, based on EUROMOD I3.0+.

	Value			Diff. w.r.t. baseline	
	Baseline	CF	COVID	CF	COVID
Gini (market income, MI)	0.4895	0.5265	0.5122	0.0370	0.0227
Gini (MI - taxes - SIC)	0.5362	0.5866	0.5677	0.0504	0.0315
Gini (MI - taxes - SIC - pensions)	0.3090	0.3441	0.3281	0.0351	0.0191
Gini (disposable income)	0.2465	0.2503	0.2451	0.0037	(− 0.0007)
Redistribution index	0.2430	0.2762	0.2663	0.0333	0.0234
Quantile share ratio (S80/S20)	3.4812	3.3706	3.4011	− 0.1106	− 0.0801
Inter-decile ratio (D5/D1)	1.8370	1.8087	1.8144	− 0.0284	− 0.0226

Values highlighted in brackets are not statistically significant (95% CI)

We can also see that this inequality-enhancing effect is almost entirely absorbed by the tax–benefit system. Taking the Gini index on disposable income as a benchmark, we observed that in the counterfactual scenario, the increase in the Gini index decreased by around 0.4pp, indicating that the standard automatic stabilizers substantially mitigated the increase in inequality, caused by COVID-19. However, we would still expect an increase in inequality in the absence of discretionary policy measures.

When we take these discretionary policy measures into account (COVID-19 scenario), this view changes. Table 1 highlights that STW schemes, as well as the one-off payment for the unemployed and children, more than offset the inequality-enhancing effect of COVID-19. In fact, we even expect the Gini index, in relation to disposable income, to drop by around 0.1pp.

Not surprisingly, redistribution increases as a result of COVID-19. This is mainly due to the progressive character of the Austrian tax–benefit system, with regard to labor market shocks (also in the absence of discretionary policy measures). The Redistribution Index increases substantially in both scenarios; however, it increases to a greater extent, when discretionary policy measures are not taken into account (CF scenario). This result is also driven by the lower impact on inequality in terms of original income in the COVID scenario.

Since it is often argued that the Gini index might not be the best measure in relation to inequality, we also analyze the impact on other inequality measures in Table 1. We can see that the income quantile share ratio, as well as the inter-decile ratio highlight the inequality-reducing character of the Austrian tax–benefit system. The reduction in both indices is lower in the COVID scenario, compared to the counterfactual scenario, because discretionary policy measures had a more significant mitigating effect than automatic stabilizer, not only in the first deciles, but also in the upper levels of the income distribution.

Table 2 highlights the impact on poverty rates across various household types. We can see a substantial increase in the AROP rate, in the absence of discretionary policy measures (CF scenario), in which the rate increases by 2.2pp from 14.8 to 17.0%. This result is mostly driven by the increase in the AROP rate for single households and households with children.

**Table 2:** The impact of COVID-19 on at-risk-of-poverty rates (%). Source: Own calculations, based on EUROMOD I3.0+.

Household type	Value			Diff. w.r.t. BL	
	Baseline	CF	COVID	CF	COVID
One adult <65, no children	24.0	28.3	25.1	4.3	(1.0)
One adult $\ge$65, no children	23.5	23.5	23.5	0.0	0.0
One adult with children	36.8	41.1	39.0	4.3	(2.1)
Two adults <65, no children	12.4	14.7	13.5	2.3	1.0
Two adults, at least one $\ge$65, no children	9.4	9.4	9.4	0.0	0.0
Two adults with one child	15.5	18.1	15.2	2.6	(− 0.3)
Two adults with two children	14.0	17.6	13.7	3.6	(− 0.3)
Two adults with three or more children	24.3	29.6	24.7	5.3	(0.4)
Three or more adults, no children	4.6	5.7	5.3	1.1	(0.7)
Three or more adults with children	14.1	14.9	14.7	0.8	(0.5)
All	14.8	17.0	15.2	2.2	0.4

Poverty line is anchored at EUR 16,086.58 (using $60\%$ of median equivalized household disposable income as the poverty line). Values highlighted in brackets are not statistically significant (95% CI)

However, discretionary policy measures (COVID-19 scenario) are able to substantially counteract this effect. In the COVID scenario, poverty slightly increases to 15.2%. We can see that the AROP rates for households with children tend to decrease in this scenario, highlighting that discretionary policy measures (such as STW schemes and one-off payments), helped to keep certain households above the poverty line, and even helped some households to cross the poverty line.^7^

### The gender component of the COVID-19 crisis

In this subsection, we will focus on the gender specific impact of the COVID-19 crisis. We use detailed administrative information by sector and gender, to identify males and females that move to STW schemes or become unemployed during the COVID-19 crisis. Additionally, we use detailed information on the duration and reduction in hours to simulate transitions in the labor market.

**Fig. 8:**
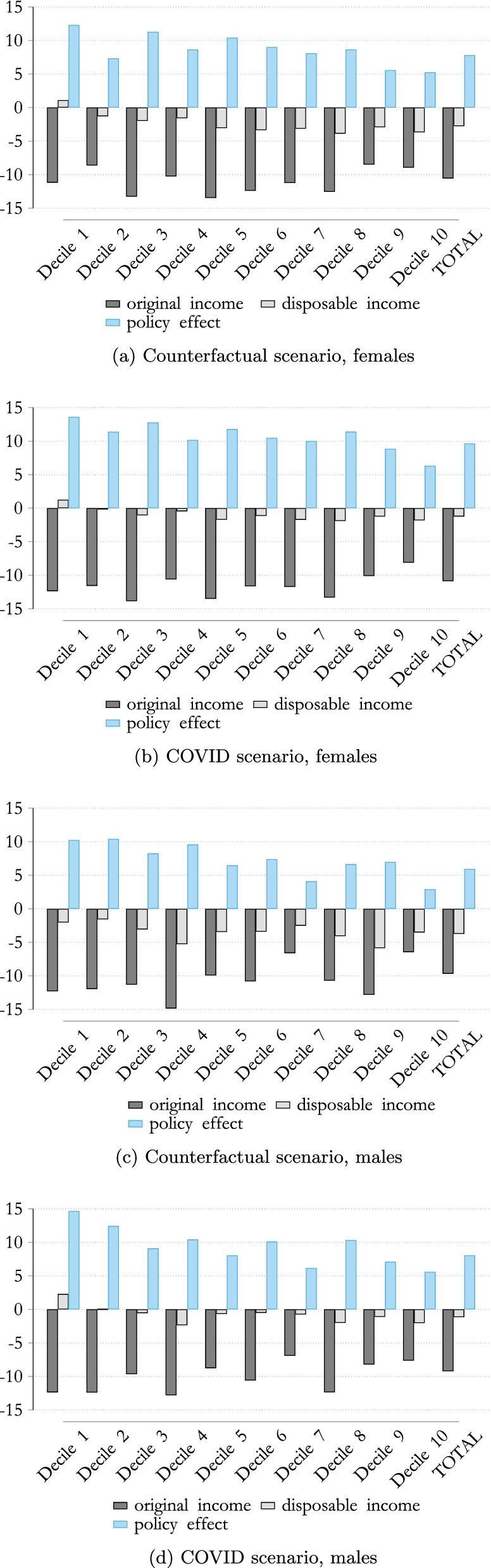
The impact of COVID-19 on household income. Note: Percentage change in equivalized original and disposable income compared to the baseline scenario by income deciles. Income deciles are based on the baseline scenario distribution of equivalized disposable income. Source: Own calculations, based on EUROMOD I3.0+.

This allows us to focus on the loss in income across the income distribution by gender. As highlighted in Fig. 8, the impact on the original (market) income during the COVID-19 crisis differs slightly. Females suffered a higher loss, on average, in terms of market income due to COVID-19, also because the sectors which were badly affected (e.g., wholesale and retail, hotel and restaurants) are characterized by a higher female employment rate. This resulted in an income loss of around 11% for females, while males lost on average 10%.

Considering the impact on disposable income, we observe that, in the absence of discretionary policy measures (counterfactual scenario), the overall mitigating effect of the tax–benefit system benefited females, and especially females at the lower level of the income distribution. This resulted in lower female wages in the lower deciles, leading to a higher net replacement rate for females under the standard tax–benefit system.^8^

The opposite holds true when discretionary policy measures are taken into account (compare Fig. 8b and d). We find a slightly greater drop in disposable income for females than for males, when taking into account discretionary policy measures. This suggests that discretionary policies measures are not able to counteract the stronger shock in the labor market income faced by women in full. Please note that for both males and females, the drop in disposable income is less than that in the counterfactual scenario.

**Fig. 9:**
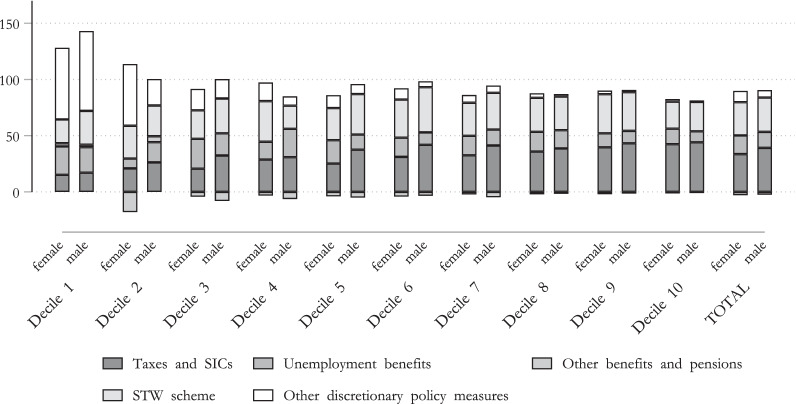
ISC in Austria by gender, COVID scenario. Note: Percentage change in equivalized original and disposable income compared to the baseline scenario by income deciles. Income deciles are based on the baseline scenario distribution of equivalized disposable income. Source: Own calculations, based on EUROMOD I3.0+.

For a more detailed impact of the mitigating effect of different policy measures, we analyze the decomposition of the ISC (*ISC*) for specific tax–benefit instruments, for both males and females. Figure 9 shows that males profit especially from taxes and STW schemes. This is mainly driven by the fact that wages of males are on average higher then wage of females.

Additionally, males move slightly more often to STW schemes than to unemployment, compared to females. Given that the replacement rate of the STW scheme is usually higher than the one of unemployment benefit, males seem to profit more from the use of this policy. However, we find that for females, other discretionary policy measures,^9^ play a more important role than for males to stabilize their income. However, one has to keep in mind that closures of schools and kindergartens have massively increased child care needs. As shown by Alon et al. (2020), this had a especially strong impact on working mothers. When analyzing these policies measures, additional gender inequality in unpaid-work is not taken into account, which, however, might influence strongly gender inequalities.

Overall, we conclude that discretionary policy measures increase the ISC substantially for both males and females. While under the standard tax–benefit rules, around 68% of the COVID-19-related income loss for females and 63% for males would be absorbed by the system,^10^ discretionary policy measures have proven their worth in strengthening the mitigating effect of the tax–benefit system in relation to the loss in income shock, especially in the case of low incomes. Overall, the *ISC* increases to 87% for females and 88% for males, indicating that both males and females profit substantially from discretionary policy measures. Additionally, we find that that for while for males, STW schemes in combination with taxes help more to stabilize their income, other discretionary policy measures (such as the one-off payments for children) are those that help more to stabilize the income of females during the COVID crisis.

## Conclusions

Using EUROMOD, the microsimulation model of the European Union, combined with detailed information relating to the Austrian labor market, we assess the impact of the COVID-19 crisis on household income in Austria. Our administrative data include information regarding STW and unemployment by gender and sector, to model labor market transitions on a micro-level. Using microsimulation techniques, we are not only able to assess the impact of the COVID-19 crisis on household income across income distribution, but also the effectiveness of discretionary policy-measures.

We demonstrate that although the impact of the COVID-19 pandemic is regressive, affecting low income households more significantly than high-income households, the automatic stabilization mechanisms of the Austrian tax–benefit system are crucial in dampening this inequality-enhancing effect. We also identify that the additional discretionary policy measures, introduced by the Austrian government (STW scheme, one-off payments for the unemployed and for children), are able to offset the income losses of poor households completely and are therefore able to completely offset the inequality-enhancing nature of the COVID-19 crisis.

Focusing on the gender differences following the COVID-19 crisis, we find that females suffered a greater loss in market income on average. The reasons are manifold. We see gender differences in movements to STW and to unemployment, as well as differences in the duration and reduction in hours. This results in an income loss in the case of females of around 11%, while males lost around 10%. When considering disposable income, we can see that the tax–benefit system (including discretionary policy measures) mitigates this loss in market income more significantly in relation to females, leading to a drop in disposable income of around 1% for both males and females.

As demonstrated, the automatic stabilizers of the Austrian tax system are crucial in mitigating against income losses for both males and females. Under the standard tax–benefit rules, around 68% of the COVID-19-related income loss suffered by females and 63% suffered by males would be absorbed by the tax–benefit system. When including discretionary policy measures, the *ISC* increases to 87% for females and 88% for males, indicating that both males and females profit substantially from discretionary policy measures. Males tend to profit slightly more from STW schemes, while for females other discretionary policy measures helps more to stabilize their incomes.

We conclude that, in Austria, discretionary policy measures have proven their worth in strengthening the mitigating effect of the tax–benefit system in relation to income losses, resulting from COVID-19, especially with regard to low incomes and in benefiting males slightly more than females. This result is mainly driven by the STW schemes. Additionally, we show that discretionary policy measures play a crucial role in checking the inequality and poverty-enhancing effect of the COVID-19 pandemic.

Our results highlight a strong protection for households against the income drop related to the COVID-19 pandemic in Austria. However, all the implemented discretionary policy measures imply substantial costs for the state budget. From a more macroeconomic perspective, the implemented policy measures are able to fully protect low income households against a drop in disposable income. Given that low-income households are typically more likely to be liquidity constrained, and that mostly households with liquidity constraints will adjust their consumption as a result of temporary income shocks in accordance to their income loss,^11^ our results indicate that the shock on total household consumption due to loss in income should be minor in Austria. Therefore, the reduction in household demand due to lockdown measures seems to be by far the main driver of the observed demand reduction during the COVID-19 pandemic. Our analysis suggests that discretionary policy measures have prevented the economy from an additional fall in household demand.

Comparing our results with similar work in other countries, we find that discretionary policy measures are especially prevalent in Austria. Compared to Germany, where Christl et al. (2021b) estimated the ACS with a similar approach, the ISC is substantially higher in Austria (around 88% in Austria vs. 81% in Germany), especially at the lower level of the income distribution. This result is not only due to a more generous STW scheme with a higher replacement rate, but is also due to other discretionary policy measures. When comparing our results with those of Cantó et al. (2021) for Spain, Belgium, the UK and Italy, only in Belgium does the tax–benefit system seem able to provide similar protection against income loss as in Austria.^12^

Our results for Austria (ISC of 88%) differ substantially to those estimated by Christl et al. (2021a) (ISC of 74%). These differences are driven by the fact that they do not include all discretionary policies such as the one-off payments for unemployed and families, which—as we have shown—play a crucial role in stabilizing household income. We find that those measures are especially important for households in the lower part of the income distribution. Our paper additionally highlights that the COVID-19 pandemic is expected to lead to an increase an inequality and poverty in the absence of policy intervention. Similar results have been found also for other pandemics in recent decades.^13^ However, as argued by Furceri et al. (2021), the observed cushioning effect of policy measures might not be long-lasting. As they have shown, the rise in inequality following after major epidemics over the last two decades (SARS, H1N1, MERS, Ebola and Zika) has been higher in episodes of greater austerity. Policy makers should keep that in mind, when the supportive measures related to COVID-19 are running out, and debt reduction will become a primary goal of policy makers.

## Figures and Tables

**Fig. 10:**
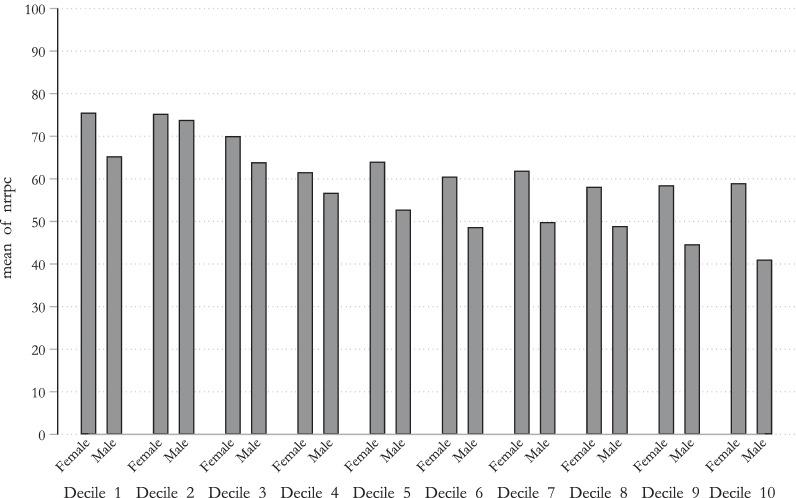
Net Replacement Rates by gender (Standard tax benefit model). Source: Own calculation, using EUROMOD I3.0+

**Fig. 11:**
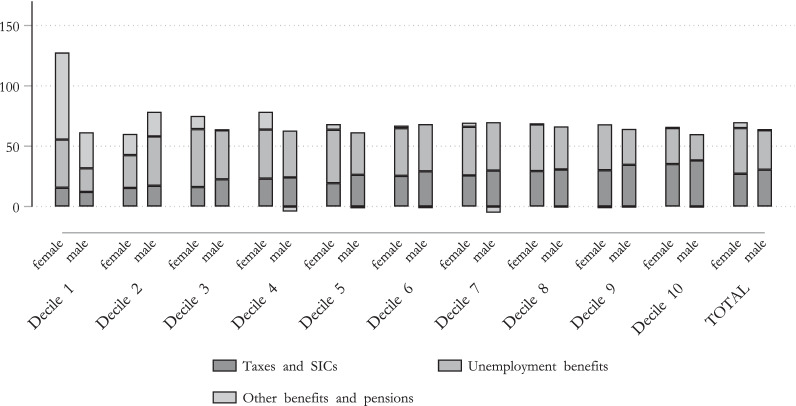
ISC in Austria by gender, counterfactual scenario. Note: Percentage change in equivalized original and disposable income compared to the baseline scenario by income deciles. Income deciles are based on the baseline scenario distribution of equivalized disposable income. Source: Own calculations, based on EUROMOD I3.0+

## Data Availability

The data that support the findings of this study are available from ESTAT and AMS (Public Employment Service Austria) but restrictions apply to the availability of these data, which were used under license for the current study, and so are not publicly available. Data are however available from the authors upon reasonable request and with permission of ESTAT.

## Appendix

See Figs. 10 and 11, Table 3.

**Table 3:** Share of working hours in short-time work on normal working hours of an employee in short-time work. Source: Own calculation, Public Employment Service Austria (AMS).

Sector	03/2020	04/2020	05/2020	06/2020	07/2020	08/2020	09/2020	10/2020	11/2020	12/2020
Agriculture, forestry and fishing	61.2%	64.4%	53.2%	45.9%	49.6%	48.9%	49.1%	45.1%	62.4%	62.4%
Mining and quarrying	29.2%	30.2%	21.9%	20.0%	30.5%	26.5%	23.0%	21.2%	24.8%	24.8%
Manufacturing	41.9%	43.4%	35.5%	29.2%	25.9%	25.8%	23.7%	25.9%	31.5%	31.5%
Electricity, gas, steam and air conditioning supply	44.7%	45.0%	28.7%	18.7%	51.1%	51.5%	46.5%	47.7%	66.8%	66.8%
Water supply, sewerage, waste management	30.3%	29.5%	22.2%	20.0%	26.6%	22.8%	33.2%	37.3%	62.7%	62.7%
Construction	40.7%	36.7%	22.1%	21.9%	31.9%	29.8%	30.5%	34.3%	39.6%	39.6%
Wholesale and retail trade, repair of motor vehicles	55.1%	57.0%	35.3%	30.4%	32.9%	29.8%	30.7%	38.5%	51.0%	51.0%
Transportation and storage	49.6%	52.1%	46.8%	39.3%	40.8%	32.6%	33.7%	40.8%	51.4%	51.4%
Accommodation and food service activities	68.7%	85.6%	64.9%	45.5%	43.2%	37.0%	40.9%	44.7%	73.6%	73.6%
Information and communication	51.8%	56.1%	51.7%	45.1%	43.3%	38.4%	37.2%	46.2%	54.2%	54.2%
Financial and insurance activities	53.0%	64.2%	56.4%	50.7%	52.5%	51.7%	53.5%	54.4%	58.2%	58.2%
Real estate activities	57.8%	66.7%	56.9%	51.4%	50.4%	44.3%	44.7%	50.5%	60.5%	60.5%
Professional, scientific and technical activities	52.9%	54.2%	47.3%	41.4%	42.7%	38.3%	37.5%	41.9%	46.3%	46.3%
Administrative and support service activities	47.0%	55.7%	45.2%	40.9%	43.4%	42.0%	42.9%	49.6%	57.1%	57.1%
Public administration and defence	84.4%	75.8%	55.2%	37.5%	54.5%	47.1%	49.9%	n.a.	71.2%	71.2%
Education	62.6%	65.9%	51.8%	36.0%	40.2%	33..0%	30.6%	51.3%	60.8%	60.8%
Human health and social work activities	59.1%	65.4%	47.9%	32.0%	36.4%	31.0%	31.9%	45.3%	47.0%	47.0%
Arts, entertainment and recreation	65.9%	74.7%	72.0%	50.5%	47.2%	41.6%	46.2%	51.4%	64.8%	64.8%
Other service activities	62.5%	76.1%	42.8%	34.4%	33.4%	29.5%	30.8%	32.0%	62.1%	62.1%
Activities of hh as employers, and others	61.6%	86.3%	77.8%	70.2%	78.5%	76.9%	87.0%	66.7%	69.5%	69.5%

Data available on 2/2021

## Abbreviations

AMS: Public employment service Austria
STW: Short-time work
LMA: Labor market adjustment
EU-SILC: EU statistics on income and living conditions
ISC: Income stabilizing coefficient
CF: Counterfactual scenario
MI: Market income
CI: Confidence interval

## Footnotes

1 See also Cantó et al. (2021), Brewer and Tasseva (2020), Christl et al. (2021a) or Christl et al. (2021b).

2 Similarly, Brewer and Tasseva (2020) conducted a study on the UK only, measuring the effects of policy responses from early 2020

3 For more information on EUROMOD, see Sutherland and Figari (2013).

4 For more details on the uprating factors, see the https://euromod-web.jrc.ec.europa.eu/usingeuromod/country-reportsAustrian EUROMOD Country Report.

5 See also the Summary Note For EUROMOD: Labour Market Adjustment Add-on (https://euromod-web.jrc.ec.europa.eu/sites/default/files/2021-02/LMAAdd-On%20Documentation.pdf).

6 10-month duration is the maximum duration in 2020, since STW schemes started in March 2020.

7 Please note that some of these results are not statistically significant

8 The Net Replacement Rate for males and females in the standard Austrian tax–benefit system (without discretionary policy measures) by gender are highlighted in Fig. 10 in the Appendix. Given the eligibility criteria (at least 52 weeks of employment in the last two years), fewer females qualify for unemployment benefits. However, on average, the net replacement rate is higher for females than for males, due to the lower level in the monthly amount of unemployment benefit.

9 We assume that the one-pay off payment for children is shared equally between partners. Since usually females with children are more likely to work part-time than males and indeed with a lower wage, in relative terms, this payment translates in a higher stabilizing effect for females.

10 See Fig. 11 in the Appendix.

11 See, e.g., Clinton et al. (2011) or Galí et al. (2007)

12 Please note that the methodology, as well as the time line (only one month is analyzed) is slightly different in the estimates of Cantó et al. (2021)

13 See, e.g., Furceri et al. (2021)
